# The Impact of Studying Brain Plasticity

**DOI:** 10.3389/fncel.2019.00066

**Published:** 2019-02-27

**Authors:** Pedro Mateos-Aparicio, Antonio Rodríguez-Moreno

**Affiliations:** Department of Physiology, Anatomy and Cell Biology, University Pablo de Olavide, Seville, Spain

**Keywords:** plasticity, development, learning and memory, recovery after injury, plasticity windows

Neural plasticity, also known as neuroplasticity or brain plasticity, can be defined as the ability of the nervous system to change its activity in response to intrinsic or extrinsic stimuli by reorganizing its structure, functions, or connections. A fundamental property of neurons is their ability to modify the strength and efficacy of synaptic transmission through a diverse number of activity-dependent mechanisms, typically referred as synaptic plasticity. Research in the past century has showed that neural plasticity is a fundamental property of nervous systems in species from insects to humans. Indeed, studies into synaptic plasticity have not only been an important driving force in neuroscience research but they are also contributing to the well-being of our societies as this phenomenon is involved in learning and memory, brain development and homeostasis, sensorial training, and recovery from brain lesions. However, despite intense research into the mechanisms governing synaptic plasticity, it is still not clear exactly how plasticity shapes brain morphology and physiology. Thus, studying synaptic plasticity is clearly still important if we wish to fully understand how the brain works.

## Historical Roots Of the Concept

The term “plastic” originates from the Latin word “plasticus,” which ultimately comes from the Greek term “plastikós” or “plastos,” originally meaning “molded, formed.” However, the roots of the modern concept of plasticity in neuroscience are still to be fully established (Berlucchi, 2002; Stahnisch and Nitsch, 2002; Jones, 2004; DeFelipe, 2006; Berlucchi and Buchtel, 2009; Markram et al., 2011). Before the nineteenth century, the brain was mainly contemplated by philosophers (Markram et al., 2011) and consequently, it was not until the late 1800s and early 1900s that the foundations were laid for modern neuroscience. In the last decade of that century, several scientists made key contributions to our modern understanding of synaptic plasticity, including the Spanish Neuroanatomist Santiago Ramon y Cajal who first defined the neuron as the anatomical, physiological, genetic, and metabolic unit of the nervous system in his Neuron Doctrine (Ramón y Cajal, 1899/1904; Shepherd, 1991; Jones, 1994a). Although less prominent, Cajal's ideas about brain plasticity were essential for the development of the concept of plasticity. In several publications and lectures between 1890 and 1894 (Ramón y Cajal, 1892, 1894a,b,c), Cajal outlined his cerebral gymnastics hypothesis, suggesting that the capacity of the brain could be augmented by increasing the number of connections (Jones, 1994b; DeFelipe, 2006). In 1893, the Italian Neuropsychiatrist Eugenio Tanzi proposed that through specific learning or practice, repetitive activity in a neuronal pathway could produce hypertrophy, thereby reinforcing the already existing connections (Berlucchi and Buchtel, 2009). The term “synapse” (previously called “junction” by Cajal) was first coined in 1897 by Foster and Sherrington in Cambridge, although unlike Tanzi, they did not elaborate on the potential relationship between synaptic plasticity and learning (Berlucchi and Buchtel, 2009; Markram et al., 2011). Later on it was Tanzi's disciple Ernesto Lugaro who suggested the chemical nature of synaptic transmission, and who formulated the link between Tanzi's theories and Cajal's ideas of neurotropism in 1906 and 1909 (Berlucchi and Buchtel, 2009). Importantly, both Tanzi and Lugaro were declared supporters of Cajal's ideas (DeFelipe, 2006; Berlucchi and Buchtel, 2009). Thus, while it may remain unclear who first coined the term plasticity, the work of Cajal undoubtedly stimulated and influenced the first theories about synapses, synaptic transmission, and synaptic plasticity.

## Multiple Forms of Synaptic Plasticity in the Twentieth Century

During the twentieth century, the question of how information is stored in the brain stimulated an enormous body of work that focused on the properties of synaptic transmission. With the publication in 1949 of “The Organization of Behavior,” the Canadian Psychologist Donald Olding Hebb articulated a theory regarding the possible neural mechanisms of learning and memory (Hebb, 1949). In his book, he declared the so-called “Hebb's postulates” that have since had an enormous influence on studies into neurophysiology. Although Donald Hebb himself admitted that he did not propose anything new, and he expresses a mixture of amusement and irritation when reference is made to the “Hebb's postulates” (Berlucchi and Buchtel, 2009), the reality is that the terms “Hebbian postulates” and “Hebbian plasticity” are now widely used in the literature. Indeed, a year before the publication of Hebb's book, the Polish Neurophysiologist Konorski (1948) postulated that morphological changes in neural connections could be the substrate of learning (Markram et al., 2011).

The first evidence linking short-term plasticity to behavioral modifications came from studies in Aplysia (Kandel and Tauc, 1965). Short-term facilitation and synaptic depression that lasts from milliseconds to minutes can be elicited by different protocols, like paired-pulse stimulation or repetitive high-frequency stimulation (Zucker and Regehr, 2002). Short-term plasticity is considered to be important in short-term responses to sensory inputs, transient modification of behavioral states, and short-term memory (Citri and Malenka, 2008). Nearly two decades after Hebb's theory first appeared, experimental evidence supporting his ideas arrived with the discovery of long-lasting potentiation in the dentate gyrus of the rabbit hippocampus (called long-term potentiation or LTP in 1975: Lømo, 2003). These findings were obtained thanks to a key technical development that occurred in parallel in Andersen's laboratory: the use of the brain slice preparation (Skrede and Westgaard, 1971). Indeed, research using the hippocampal slice preparation has continued to enhance our understanding of synaptic plasticity over the years. Another form of long-term plasticity, long-term depression, or LTD, was first proposed in 1977 (Lynch et al., 1977). These advances, along with the development of intracellular recordings in brain slices and patch-clamp techniques, led to the identification of different forms of short- and long-term plasticity at distinct synapses across the brain. For example, in the 80s NMDA receptors were shown to be involved in synaptic plasticity (Herron et al., 1986) and postsynaptic AMPA receptors were seen to be important during LTP (Kauer et al., 1988), important findings in our efforts to understand synaptic plasticity.

In the last decade of the twentieth century, the importance of the relative timing of action potentials generated by pre- and postsynaptic neurons at monosynaptic connections was shown when measured in pairs of cortical neurons (Markram et al., 1997, 2011), representing a new framework to study plasticity. The elegance and simplicity of this experimental paradigm, also called spike-timing-dependent plasticity (STDP), attracted the attention of the neuroscientific community. However, further studies into the rules governing STDP in different types of neurons and synapses revealed a much more complex landscape (Markram et al., 2011). Moreover, another form of persistent synaptic plasticity was suggested at that time, called metaplasticity (Abraham and Bear, 1996). Metaplasticity, also known as “the plasticity of synaptic plasticity,” is a phenomenon that involves the activity-dependent changes in neuronal function that modulate synaptic plasticity. The role of metaplasticity is not yet clear but it may serve to maintain synapses within a dynamic range of activity, allowing synapses and networks to respond to a changing environment. At the end of the twentieth century, a new form of plasticity that operates over longer time scales was discovered, called homeostatic plasticity (Turrigiano et al., 1998; Turrigiano and Nelson, 2004). Homeostatic plasticity involves a number of phenomena that balance the changes in neural activity to maintain homeostasis over a wide range of temporal and spatial scales (Turrigiano, 2012). The best studied example of homeostatic plasticity is known as synaptic scaling (Turrigiano et al., 1998), which allows neurons to detect changes in their own firing rates through a set of calcium-dependent sensors that then regulate receptor trafficking, thereby increasing or decreasing the number of glutamate receptors at synaptic sites (Turrigiano, 2012). The relationship between STDP and homeostatic plasticity is not well-understood and it is currently an interesting area of research (Watt and Desai, 2010).

In parallel with activity-dependent changes in synaptic strength and efficacy of synaptic transmission, structural modifications of axonal, dendritic branches, and spine morphology occurs, a phenomenon called structural synaptic plasticity. In particular, different studies have correlated bidirectional structural spine changes with activity-dependent synaptic plasticity, i.e., increased spine size upon LTP (Engert and Bonhoeffer, 1999; Matsuzaki et al., 2004) or spine shrinkage upon LTD (Nagerl et al., 2004; Zhou et al., 2004). Nowadays, *in vivo* two-photon imaging techniques combined with electrophysiological recordings are instrumental in order to clarify the relationship between functional-structural synaptic plasticity and behavior. For example, spine formation has been observed following successful reaching task related with motor memories (Xu et al., 2009), whereas spine loss has been associated with fear conditioning (Lai et al., 2012).

## Challenges and Perspectives in Plasticity Research

Numerous important findings on the mechanisms of LTP and LTD, such as the importance of NMDA receptors, their timing dependence, the locus of expression or the molecular mechanisms underlying LTP and LTD fueled intense debate by the end of the twentieth century (Madison et al., 1991; Malenka and Nicoll, 1993, 1999; Malenka and Bear, 2004). Furthermore, the discovery of STDP at the beginning of this century, generated interest in the influence of timing and frequency on the parameters required to induce synaptic plasticity (Lisman and Spruston, 2005, 2010; Markram et al., 2011). This was in part because traditional forms of plasticity are provoked with protocols based on stimulation frequencies that are sometimes far from physiological, and they are therefore unlikely to occur *in vivo*. On the other hand, although other forms of dendritic depolarization rather than the back-propagated action potential may be sufficient to induce STDP, there is an increasing body of evidence showing that STDP can be induced by just one timely generated single action potential relative to the EPSP (Rodríguez-Moreno and Paulsen, 2008; Andrade-Talavera et al., 2016; Pérez-Rodríguez et al., 2018). Indeed, most of the rules and properties of STDP have been defined *in vitro*, yet STDP was detected *in vivo* (Markram et al., 2011). Thus, a key future challenge will be to determine the mechanisms, rules and roles of STDP *in vivo* (Schulz, 2010). In this regard, it will be important to define the precise influence of neuromodulators on STDP (Pawlak et al., 2010). In addition, it will be necessary to develop a unitary mechanistic framework that simplifies and explains the tremendous variability in the properties of STDP in different brain regions and synapses. This will be aided considerably be clearly establishing the expression and role of presynaptic NMDA receptors (Sjöström et al., 2003; Rodríguez-Moreno and Paulsen, 2008; Abrahamsson et al., 2017; Costa et al., 2017; Bouvier et al., 2018), particularly in light of the proposed metabotropic activity of these receptors (Nabavi et al., 2013, 2014; Dore et al., 2016, 2017). Finally, more detailed studies will be required to define the recently demonstrated role of glial cells in synaptic plasticity (e.g., Perea and Araque, 2007; Navarrete et al., 2012; Allen and Lyons, 2018), determining the exact role of glial cells in plasticity at different ages.

Synaptic plasticity is intrinsic to the development and function of the brain, and it is essential for learning and memory processes. In addition, the time windows for plasticity exist during development shape the connections in the brain and its activity (Hensch, 2004; Rodríguez-Moreno et al., 2013; Pérez-Rodríguez et al., 2018). Thus, investigating how synaptic plasticity occurs and how it is modified during specific developmental time windows will provide key information as to how the brain develops. Moreover, better understanding how synaptic modifications take place during learning and memory, and/or development, may help shape, and improve the efficacy of current protocols at early stages of academic learning. Furthermore, the translational relevance of animal studies of synaptic plasticity must be further clarified in the future. Studies in human tissue indicate that synaptic plasticity of human synapses is a candidate mechanism for learning and memory, although direct evidence of the actual cellular mechanism is lacking (Mansvelder et al., 2019). As observed in animal studies, activity-dependent, Hebbian-like synaptic changes can be induced in the human brain *in vivo*, although with differences in the specific plasticity rules (Mansvelder et al., 2019). Current electrophysiological and imaging techniques commonly used in animal models can be used for *in vitro* experiments with human tissue from dissected patients. However, a major challenge for the future is to study synaptic plasticity in the human brain *in vivo*. To this end, non-invasive techniques like transcranial magnetic stimulation (TMS) may represent a step forward (Polania et al., 2018).

On a different note, plasticity is also a phenomenon that aids brain recovery after the damage produced by events like stroke or traumatic injury. Indeed, the ability to manipulate specific neuronal pathways and synapses has important implications for therapeutic and clinical interventions that will improve our health. Promising therapies like deep brain stimulation, non-invasive brain stimulation, neuropharmacology, exercise, cognitive training, or feedback using real-time functional magnetic resonance (Cramer et al., 2011), are all based on our current understanding of brain plasticity and they are the subject of intense research for different pathologies. A better understanding of the mechanisms governing neuroplasticity after brain damage or nerve lesion would help improve patient's quality of life, eventually saving costs to National Health Systems worldwide. Therefore, the study of synaptic plasticity has clear consequences that reach beyond the research environment. Increasing our understanding of how learning and memory processes are modified during development, and of how the brain modifies its activity and recovers after damage, should be considered in some depth by policy makers. In the light of the above, such efforts are likely to provide social benefits in the spheres of Healthcare and Education, thereby aiding long-term socio-economic planning.

## Author Contributions

All authors listed have made a substantial, direct and intellectual contribution to the work, and approved it for publication.

### Conflict of Interest Statement

The authors declare that the research was conducted in the absence of any commercial or financial relationships that could be construed as a potential conflict of interest.
